# Can Milking-Like Effect Be the First Clue of a Ventricular Free Wall Rupture?

**DOI:** 10.7759/cureus.23042

**Published:** 2022-03-10

**Authors:** Ramses Ramirez Damera, Alexander Kong, Muhammad I Khan, Hiren Patel, Aamir Javaid

**Affiliations:** 1 Internal Medicine Residency, University of Central Florida College of Medicine, Orlando, USA; 2 Cardiovascular Disease, Saint Louis University School of Medicine, St. Louis, USA; 3 Cardiology, University of Central Florida College of Medicine, Orlando, USA

**Keywords:** cardiogenic shock, cardiac tamponade, myocardial bridge, myocardial rupture, myocardial infarction complication

## Abstract

Ventricular free wall rupture (VFWR) is a catastrophic complication of myocardial infarction that poses an imminent surgical emergency. Early recognition is essential as it can expedite the process for a life-saving surgical intervention. We present a case of an acute left VFWR resulting from an underlying myocardial infarction which showed a “milking-like effect” during diagnostic angiography. “Milking-like effect” is an angiographic phenomenon typically seen in myocardial bridging, which occurs due to the compression of the intramyocardial coronary segments during systole. The presence of this phenomenon is believed to occur due to the extrinsic compression of the coronary by the evolving hemopericardium.

## Introduction

Ventricular free wall rupture (VFWR) is a known catastrophic complication of myocardial infarction [1]. It is associated with an extremely high mortality risk and poses an imminent surgical emergency with poor outcomes [2]. Risk factors associated with VFWR include the use of thrombolytic agents, female gender, and large areas of ischemia [3-6]. The underlying pathogenesis is directly related to the weakening or thinning of the architecture as a result of myocardial cell demise, which occurs regardless of whether reperfusion is achieved after the acute ischemic event [7]. Recognizing this complication in the absence of signs of cardiogenic shock remains a very difficult task for most clinicians as there are no risk stratifying tools or clinical clues to help identify those who are at a higher risk and would benefit from further assessment or monitoring. In 2016, Bastante et al. published a report of two patients who presented with ST-segment elevation myocardial infarction (STEMI) and had a “milking-like effect” during emergent angiography. Interestingly, both cases became hemodynamically unstable promptly after catheterization and were then found to have a VFWR; unfortunately, none of the patients survived this tragic complication [8]. We present the case of an acute left VFWR resulting from an underlying myocardial infarction that showed a “milking-like effect” during diagnostic angiography.

## Case presentation

A 66-year-old gentleman was brought to the emergency department due to ongoing chest pain that had started earlier during the day. The chest pain was described as constant, non-exertional, without any worsening or relieving factors. The patient also reported having a single syncopal episode on the morning of the presentation; however, the family at bedside was unable to provide further details of the incident. His medical history was significant for severe aortic regurgitation and aortic root dilatation for which he had undergone a bovine aortic valve replacement (AVR) five years before presentation. His other chronic conditions included hypertension and benign prostatic enlargement.

Of significance, the patient had a similar episode of chest pain two days prior and at that time he was taken to another emergency department. An electrocardiogram (ECG) done at that time was normal, according to the patient and family members, so he was sent home but instructed to return for further evaluation if the pain recurred.

**Figure 1:**
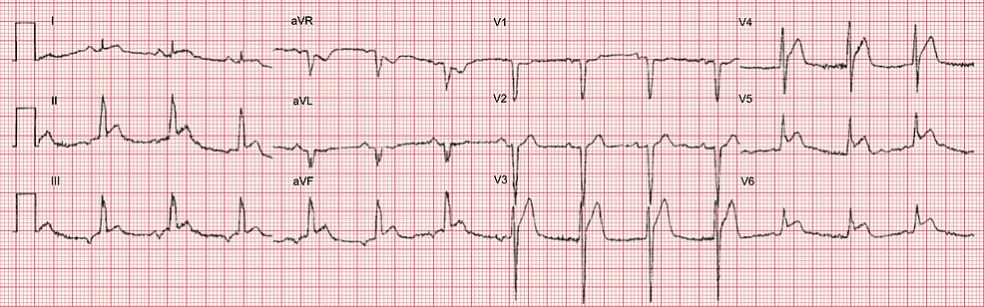
Initial electrocardiogram revealing lateral precordial lead ST-segment elevations (V4, V5, and V6) indicating lateral STEMI. STEMI: ST-segment elevation myocardial infarction

Upon presentation to our facility, physical examination revealed an elderly gentleman in mild distress due to chest pain, heart sounds were diminished, but there were no added rubs, gallops, or murmurs identified. The remainder of the examination was unremarkable. Initial vital signs showed a blood pressure of 86/55 mmHg, heart rate of 101 beats/minute, respiratory rate of 16 breaths/minute, and normal oxygen saturation. Initial ECG revealed a STEMI in the lateral leads (Figure 1), which led to the activation of the STEMI protocol. He also underwent a chest radiograph that revealed mild cardiomegaly, but no other acute cardiopulmonary findings. Relevant laboratory findings on arrival included a hemoglobin 10.8 g/dL, platelets 155,000/mm^3^, partial thromboplastin time 31.8 seconds, and international normalized ratio 1.3. Troponin I was 11.51 ng/mL, and brain natriuretic peptide was 1,464 pg/mL.

**Figure 2:**
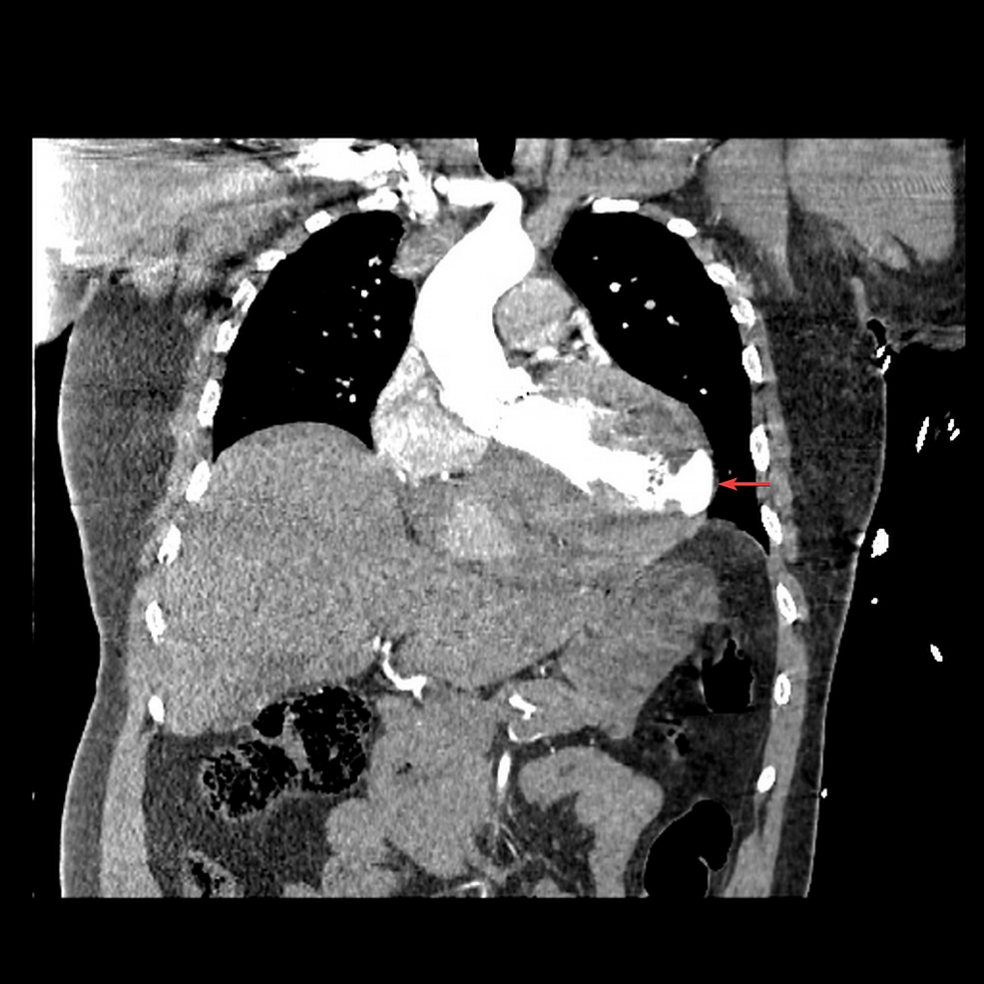
Coronal view of computed tomography with angiography of the chest showing a ventricular free wall rupture with extravasation of contrast from the left ventricle to the pericardial space (red arrow).

**Figure 3:**
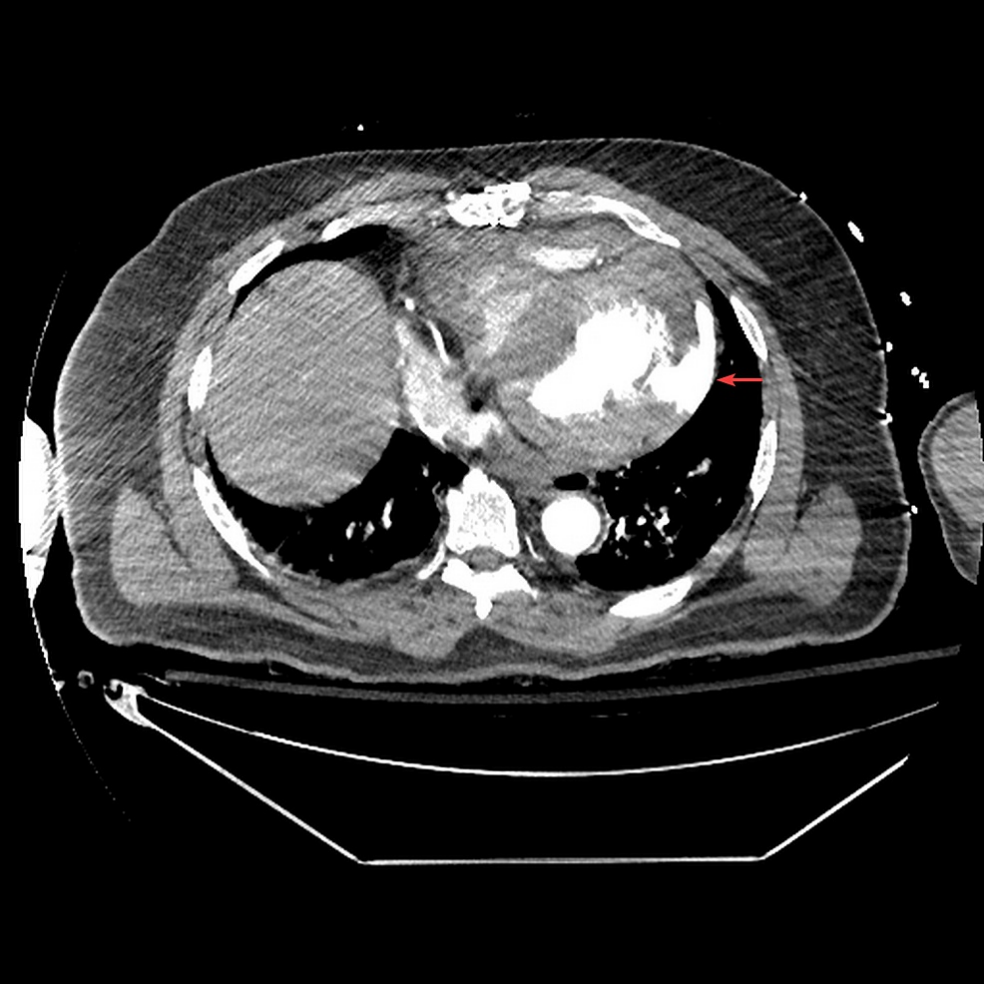
Transverse view of computed tomography with angiography of the chest showing a ventricular free wall rupture with extravasation of contrast from the left ventricle to the pericardial space (red arrow).

A computed tomography with angiography (CTA) of the chest was performed as the patient was being taken to the catherization laboratory. This was done because aortic dissection was on the differential, given his history of AVR, and for that reason intravenous heparin was not initiated. During the cardiac catheterization, the CTA results were called in revealing a lateral free wall rupture with hemopericardium (Figures 2, 3).

It was then decided to limit the angiography to the preliminary coronary anatomy findings so far observed, and the cardiothoracic surgery team was made aware of the critical results. Interestingly, the preliminary evaluation revealed a cut-off of the second marginal, most likely secondary to left ventricular perforation, but the rest of the coronaries were not obstructed (Video 1). To our surprise, a “milking-like effect” was seen at the mid to distal portion of the left anterior descending coronary artery, with almost complete disappearance during systole (Videos 2, 3).

**Video 1:** Cardiac angiography showing a cut-off of flow at the second marginal branch of the left circumflex coronary artery (red arrow), which was anatomically congruent with the area of free wall rupture.

**Video 2:** Cardiac angiography of the mid left anterior descending coronary artery revealing an intermittent filling pattern (red arrow), also known as a coronary milking-like effect.

**Video 3:** Cardiac angiography of the mid left anterior descending coronary artery revealing an intermittent filling pattern (red arrow), also known as a coronary milking-like effect.

Shortly after the angiography, the patient became hemodynamically unstable and subsequently went into pulseless electric activity (PEA). Cardiopulmonary resuscitation was started by the critical care team that was able to intubate the patient and achieve return to spontaneous circulation (ROSC) within 10 minutes. After achieving ROSC, the patient was emergently transported to the operating room; however, before being able to arrive there, he became hemodynamically unstable and subsequently went into PEA once again. Despite all attempts made by the critical care and surgical teams, he finally succumbed to his illness.

## Discussion

VFWR remains a difficult entity to diagnose early in its disease course as patients can present with minimal nonspecific symptoms such as chest pain or syncope or they can present in cardiogenic shock [9]. This can delay the recognition of the need for emergent surgical intervention and repair. VFWR is most commonly seen as a post-myocardial infarction complication with either acute or subacute presentation [10,11].

The “milking-like effect” is an angiographic phenomenon that is typically seen in myocardial bridging, which is a congenital disease that results in partial or total intramural coronary distribution. In this subset of patients, the “milking-like effect” occurs from the compression of the intramyocardial coronary segments during systole. This leads to an intermittent filling pattern, depending on the cardiac cycle, which is a hallmark finding of myocardial bridging [12]. “Milking-like effect” has been previously described in association with left ventricular pseudoaneurysms and true aneurysms [13-15]. This angiographic phenomenon is thought to occur because of the acquired dilation of the ventricle, leading to extrinsic compression of the coronary arteries during systole. Bastante et al. were the first to report the presence of a “milking-like effect” in the setting of VFWR and proposed that this angiographic finding could be an early clue of an evolving VFWR.

Our case was unique in that the patient had a CTA before cardiac angiography, which allowed us to recognize the VFWR before hemodynamic compromise. Moreover, despite not being a thorough angiographic evaluation, we were able to grossly assess anatomy and cardiovascular hemodynamics, which is seldom reported in these patients. We also believe that this phenomenon is the result of a temporarily sealed VFWR, with extrinsic compression of the coronary by the evolving hemopericardium.

## Conclusions

The presence of a “milking-like effect” may be an early clue of a developing VFWR. As such, when seen in association with an acute myocardial infarction, it should prompt providers to obtain further imaging as it can be a life-saving decision.
